# Steps towards implementation of protocolized dose reduction of adalimumab, etanercept and ustekinumab for psoriasis in daily practice

**DOI:** 10.1080/09546634.2023.2186728

**Published:** 2023-03-13

**Authors:** L.S. van der Schoot, J.J. Janssen, M.T. Bastiaens, A. de Boer-Brand, C. Christiaansen-Smit, D.N.H. Enomoto, R. Hovingh, R.A. Tupker, M.M.B. Seyger, L.M. Verhoef, J.M.P.A. van den Reek, E.M.G.J. de Jong

**Affiliations:** a Department of Dermatology, Radboud University Medical Center, Nijmegen, The Netherlands; b Department of Dermatology, Elisabeth-TweeSteden Ziekenhuis, Tilburg, The Netherlands; c Department of Dermatology, St Antonius Ziekenhuis, Nieuwegein, The Netherlands; d Department of Dermatology, Dermatologisch Centrum Isala, Zwolle, The Netherlands; e Department of Rheumatology, Sint Maartenskliniek, Nijmegen, The Netherlands; f Radboud University, Nijmegen, The Netherlands

**Keywords:** Psoriasis, biologics, dose reduction, implementation

## Abstract

**Background:**

Dose reduction (DR) of adalimumab, etanercept and ustekinumab has proven to be (cost-)effective in psoriasis patients with low disease activity. Further implementation is needed to establish application of DR for eligible patients.

**Objectives:**

To evaluate the implementation of protocolized biologic DR in daily practice.

**Methods:**

A pilot implementation study was performed in 3 hospitals during 6 months. By combining education and protocol development, involved healthcare providers (HCPs) were directed toward the adoption of protocolized DR. DR of adalimumab, etanercept, and ustekinumab was achieved by stepwise injection interval prolongation. Implementation outcomes (fidelity, feasibility) were assessed. Factors for optimizing implementation were explored in interviews with HCPs. Uptake was measured in patients by chart review.

**Results:**

The implementation strategy was executed as planned. Implementation fidelity was less than 100% as not all provided tools were used across study sites. HCPs indicated the feasibility of implementing protocolized DR, although time investment was needed. Identified additional factors for successful implementation included support for patients, uptake of DR into guidelines, and supportive electronic health record systems. During the 6 months intervention period, 52 patients were eligible for DR of whom 26 (50%) started DR. The proposed DR protocol was followed in 22/26 patients (85%) on DR.

**Conclusion:**

Additional staff for support, extra time during consultations, education on DR for HCPs and patients, and effective tools such as a feasible protocol can lead to more patients on biologic DR.

## Introduction

Dose reduction (DR) of the highly effective but expensive biologics for psoriasis could prevent overtreatment and result in more efficient use of these drugs. Previous studies showed that DR of the biologics adalimumab, etanercept, and ustekinumab is (cost-)effective in a substantial amount of psoriasis patients with low disease activity, without losing disease control (1,2). By following a disease-activity-guided DR protocol, the lowest effective dose can be achieved and timely actions can prevent the loss of adequate treatment responses.

Biologic DR is to some extent already performed in clinical practice. Practice is however heterogeneous, which may not always lead to safe and effective DR (3–5). Recommendations or guidelines are lacking. In order to standardize practice and establish the application of biologic DR for eligible patients, further implementation of protocolized DR into clinical practice is needed.

Factors that might influence the application of biologic DR in daily practice were previously explored by assessing the attitudes and behavior of dermatologists in national and international settings (3,4,6). Insight into factors influencing uptake of innovations into practice is important, as establishing the effectiveness of clinical innovations or incorporating innovations into guidelines does not guarantee uptake (7,8). Previously reported barriers to application of biologic DR among dermatologists were the belief that patients are not willing to reduce their dose, forgetting to discuss DR, lack of time, fearing reduced effectiveness, and lack of guidelines or scientific evidence (3,6). Our previous evaluation of a DR strategy of adalimumab, etanercept and ustekinumab in daily practice showed that performing such a strategy in daily practice was possible, but required some time investment (9). Based on these previously identified barriers, implementation of biologic DR could be improved by targeting healthcare providers’ (HCPs) behavior with the provision of education and guidance.

Implementation research covers the field of research focusing on enhancing the uptake of research findings or innovations into routine practice (8). For better uptake of innovations, tailored implementation strategies can be developed which include methods or techniques used to enhance the adoption, implementation and/or sustainability of innovations into practice (10,11). For the development of such strategies, different frameworks or checklists exist of which constructs relevant to the specific context can be selected (12–16). These theoretical frameworks help to identify the most important barriers to change within a specific context, to develop a strategy with components targeting these barriers, and to select outcomes and guide data collection for appropriate evaluation of implementation processes.

In the present study, we conducted a pilot implementation study in a national setting. A multi-component implementation strategy was developed, which targeted several previously identified barriers to the application of DR among healthcare providers (HCPs) as described above. Components were based on a theoretical framework for effective implementation (13). Combining education, feedback, and development of local protocols, involved HCPs in 3 general hospitals were directed toward the adoption of protocolized DR of the biologics adalimumab, etanercept, and ustekinumab for patients with psoriasis. We aimed to evaluate the implementation process and explore possible factors for optimizing the implementation of biologic DR.

## Materials and methods

A pilot implementation study was performed in 3 general hospitals in the Netherlands during 6 months. Involved HCPs were directed toward adoption of protocolized DR of the biologics adalimumab, etanercept, and ustekinumab for psoriasis, using a multicomponent implementation strategy. DR was achieved by injection interval prolongation in two steps, leading to 67% and subsequently to 50% of the standard dose. Evaluation comprised two parts: 1) process evaluation focusing on fidelity and feasibility of the implementation strategy, and 2) effect evaluation comprising an explorative evaluation of the actual innovation uptake measured in patients (e.g., numbers of patients on biologic DR). Both quantitative and qualitative methods were utilized in order to provide broad insights into the implementation process. All used outcomes are described below.

This study was conducted according to the ICH GCP guidelines and the principles of the Declaration of Helsinki. The need for ethical approval was waived by the medical ethical committee Arnhem-Nijmegen (2021-8164). Local approval from the participating hospitals was requested. Written informed consent was obtained from all interview participants (part 1) and patients (part 2). Reporting follows the Standards for Reporting Implementation Studies (StaRI) statement (17), and the Standards for Reporting Qualitative Research (SRQR) (18).

### Study setting and participants

Three hospitals were selected for participation. Eligible hospitals were general dermatology outpatient clinics with experience in treating psoriasis patients with biologics, without previous participation in clinical studies regarding protocolized DR. Participating hospitals were selected through the network of the Radboudumc and included Elizabeth-TweeSteden hospital Tilburg, Isala hospital Zwolle, and St. Antonius hospital Nieuwegein.

The implementation strategy was directed toward HCPs rather than directly at patients. Patient representatives from the national psoriasis patient association were however present within the overarching project team. This team also consisted of clinicians, researchers, and representatives from the Dutch dermatologists association, and was involved in steering different projects on biologic DR.

### Dose reduction protocol

**Figure 1.**
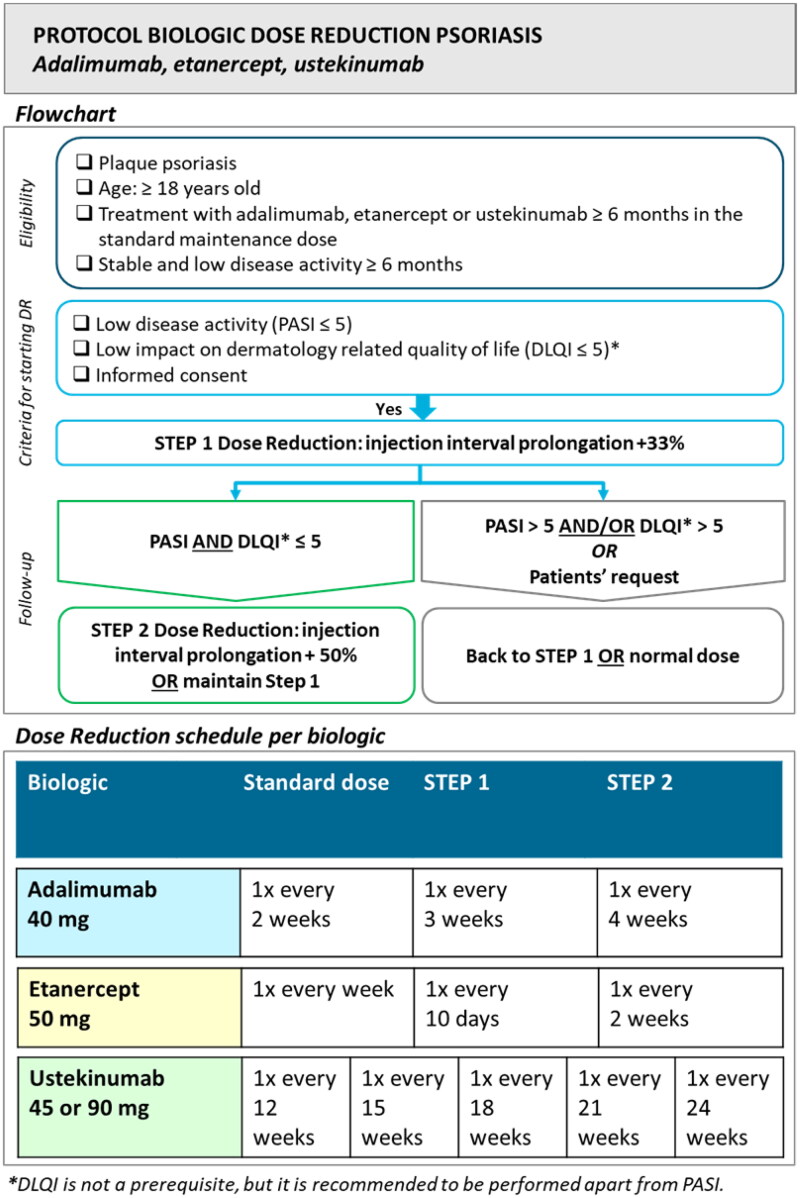
Dose reduction protocol. Abbreviations: PASI: Psoriasis Area and Severity Index; DLQI: Dermatology Life Quality Index.

The studied innovation (e.g., protocolized biologic DR) focused on psoriasis patients with stable low disease activity for at least 6 months who were treated with adalimumab, etanercept, or ustekinumab in the standard dose for at least 6 months. The DR protocol was based on a previously conducted randomized trial (19). DR was achieved by injection interval prolongation to 67% and subsequently to 50% of the original dose when Psoriasis Area and Severity Index (PASI) (or description of disease activity) and preferably Dermatology Life Quality Index (DLQI) remained low (scores ≤5). DLQI was incorporated as a flexible criterion to improve the feasibility of the protocol. In case of scores >5 and/or at patients’ request, the previous effective dose or normal dose was resumed. See Figure 1 for the used DR protocol. Visit schedules were performed according to the usual practice in participating hospitals.

### Implementation strategy

**Figure 2.**
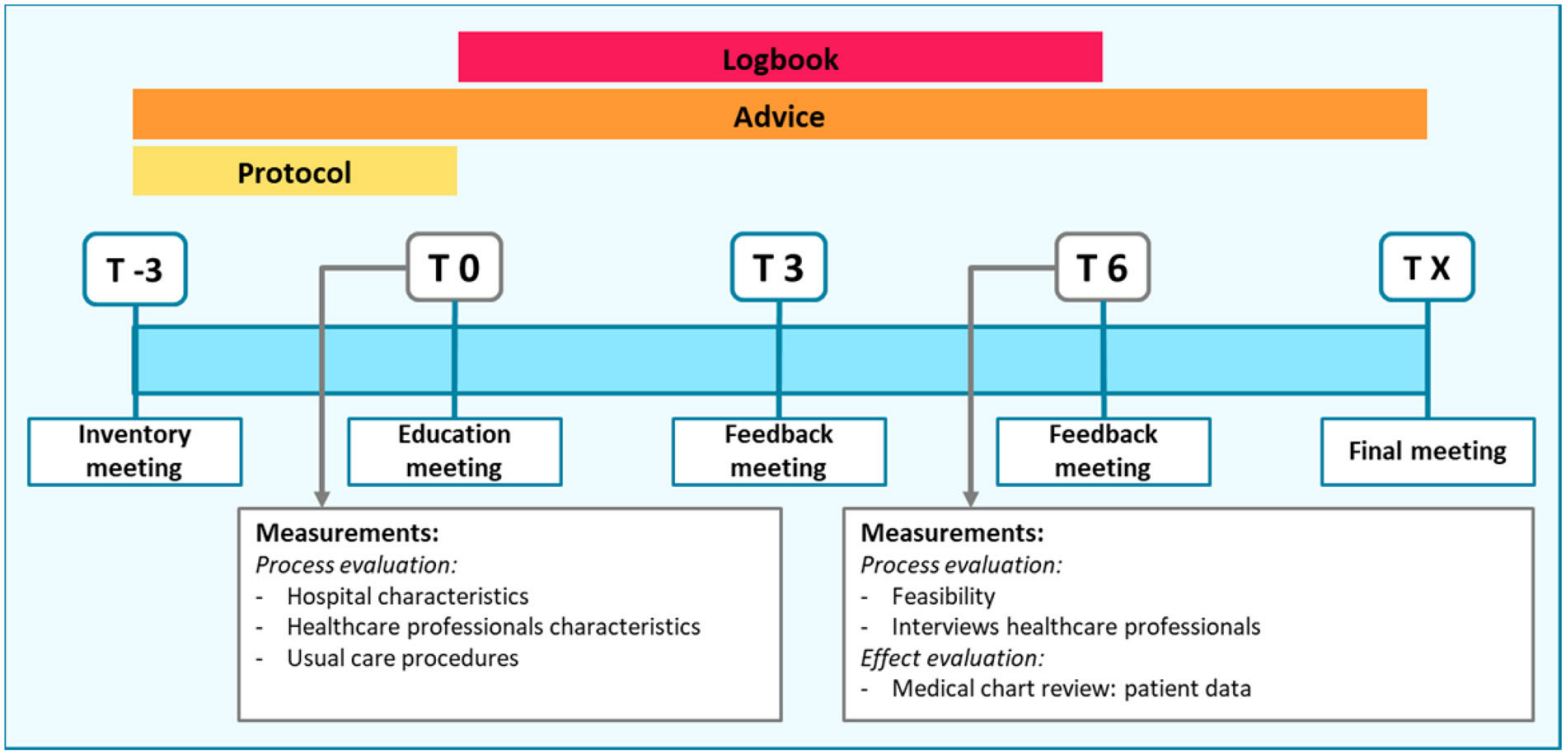
Summary and timeline of the implementation strategy. Abbreviations: T: time in months.

The used multicomponent implementation strategy combined education, feedback, and development of local protocols. This strategy was developed and provided by researchers (LvdS, JvdR, EdJ) with experience in performing protocolized biologic DR. The detailed content of the implementation strategy is presented in supplementary Table S1. Components of the strategy were based on the implementation framework of Flottorp et al. (13). The strategy contained characteristics of an academic detailing approach, in which trained HCPs visit other HCPs to provide evidence-based information, tailored advice, and support according to the specific situation (20). The strategy consisted of 5 meetings together with the development of local protocols and tools for the assistance of HCPs. Involved HCPs received a presentation with background information on biologic DR by the researcher, and local workflows were discussed. Tools included protocols, summary cards (Figure 1), patient information leaflets (Figure S1), and standardized texts for administrating DR procedures in patients’ electronic health records. Tools were tailored to local situations. Feedback meetings were provided on group level split per participating hospital, and consisted of discussing local workflows, advice, and HCPs feedback on the implementation process. Outcomes of the effect evaluation (part 2) were provided at the final meeting. A summary of the planned strategy in each hospital is depicted in Figure 2.

**Table 1.** Baseline characteristics of participating hospitals and usual care procedures.

	Hospital 1	Hospital 2	Hospital 3
Hospital type	Non-academic	Non-academic	Non-academic
PASI performed	Yes	No	Yes
PASI calculation available in electronic health record	Yes	No	No
DLQI performed	Yes	No	Yes
DLQI calculation available in electronic health record	Yes	No	No
DR performed	Yes	Yes	Yes
Time per outpatient visit (dermatologist), minutes	10	10	10
Time per outpatient visit (nurse/nurse practitioner), minutes	20	15	30
Mean number of outpatient visits for psoriasis patients on biologic treatment per year	3	2	3
Total number of patients per biologic			
Adalimumab	29	152	75
Etanercept	6	11	12
Ustekinumab	179	37	78

Abbreviations: PASI: psoriasis area and severity index; DLQI: dermatology life quality index; DR: dose reduction.

### Outcomes and data collection

#### Part 1. Process evaluation

#### Quantitative

Several outcomes were selected in order to evaluate the implementation process. These implementation outcomes were drawn from different implementation theories (11,16,21,22). First, sample characteristics (hospital characteristics, usual care procedures, and characteristics of involved HCPs) were assessed at baseline (T0, Figure 2). Second, implementation fidelity was measured. Implementation fidelity captures the degree to which an implementation strategy is delivered as prescribed (11,22). It was registered by using a logbook whether the strategy was executed according to schedule and what tools were developed and actually used. Third, the feasibility of the implementation process was evaluated. At the month 3 and 6 feedback meetings (T3, T6, Figure 2), the experiences of involved HCPs were asked by short questions covering feasibility and timeinvestment. Data were pseudonymized and collected using a web-based data management system (www.castoredc.com).

#### Qualitative

Involved HCPs participated in semi-structured qualitative interviews (T6, Figure 2) in order to evaluate feasibility of the implementation process, and to explore possible factors for optimizing implementation of biologic DR. An interview topic guide was developed by the researchers (LvdS, JJ), and focused on general views toward the implementation strategy, acceptability, feasibility, and complexity of the intervention. Interviews were held by telephone by one researcher (LvdS). A sample size was not predefined, but all HCPs involved in the study were invited to participate.

#### Part 2. Effect evaluation

Actual uptake or adoption of the implemented innovation was measured in patients using data from medical charts. As this was a pilot study, we performed an explorative analysis of the percentage of patients starting biologic DR during the intervention period (“post-intervention”), as well as the percentage of patients eligible for DR but who did not initiate DR during the intervention period. It was assessed if the proposed DR protocol was followed and if outcome measures (PASI, DLQI) were used. Here, the main goal was to assess uptake of the implemented DR protocol and not to evaluate DR effectiveness or safety in patients. Data of patients on adalimumab, etanercept, and ustekinumab who provided written informed consent were collected in 2 participating hospitals (Elizabeth-TweeSteden hospital Tilburg and Isala hospital Zwolle) through retrospective medical chart review. One hospital was excluded from this analysis as local arrangements could not be made. Included patients were not necessarily candidates for biologic DR, as all patients using adalimumab, etanercept, or ustekinumab were invited to participate. Collected patient characteristics included age, sex, body mass index (BMI), type of biologic, duration of psoriasis until the start of biologic, psoriasis subtype, comorbidities such as psoriatic arthritis (PsA), history of previous biologic use, concomitant systemic psoriasis treatment, baseline PASI and DLQI and previous DR. Baseline characteristics were collected from the last available visit before the start of the intervention period with a window of one year. During the intervention period, collected data included type of biologic, biologic dose, disease activity measures, and DR or biologic discontinuation with corresponding reasons upon availability. Here, data of the patients” most closely situated visit to the end of the 6 months intervention period was used. Data were pseudonymized and collected using a web-based data management system (www.castoredc.com).

### Analysis

#### Part 1. Process evaluation

Quantitative data were summarized. Qualitative data from the interviews were analyzed using inductive thematic analysis (23), with ATLAS.ti software. Interviews were audio-recorded, transcribed verbatim, checked for accuracy against the audio recordings and pseudonymized. Two researchers (LvdS, JJ) analyzed the first three transcripts independently (open coding). All other transcripts were coded by one researcher (JJ) and reviewed by another researcher (LvdS). Differences were discussed until a consensus was reached. Based on the initial codes, the next transcripts were systematically coded (axial coding). Newly identified themes were added to the code list, using an inductive approach without trying to fit data into any predetermined category. Analysis resulted in a list of themes influencing the implementation of biologic DR. Corresponding quotes were selected from the interviews, and were translated into English. Final results were presented to participants in order to check for accuracy.

#### Part 2. Effect evaluation

Quantitative data were analyzed using descriptive statistics in SPSS Statistics 25 (IBM, Armonk, NY, USA). Baseline patient and treatment characteristics were summarized. Depending on the type of variable and its distribution, descriptive statistics are presented as percentages with absolute numbers, means with 95% confidence intervals (CI) or medians, and interquartile ranges (IQR). Proportions of patients on lowered dosages, and proportions of patients eligible for DR during the intervention period according to the proposed protocol (plaque psoriasis, sustained low disease activity ≥6 months, use of the standard maintenance dose ≥6 months, low impact of psoriasis on patients’ dermatology-related quality of life, no failed previous DR attempt) were calculated. Reasons for DR ineligibility were summarized. It was assessed if the proposed DR protocol was followed based on used dosing schedules and whether criteria were met. Additionally, it was counted if PASI and DLQI were measured.

## Results

The study took place in 3 regional outpatient dermatology departments. Inventory meetings were held in April 2021 (hospitals 1 and 3) and May 2021 (hospital 2). The intervention period of 6 months (e.g., actual implementation of the DR protocol) started after education meetings in June 2021 (hospital 2) and July 2021 (hospitals 1 and 3).

### Part 1. Process evaluation

#### Sample characteristics

Table 1 shows the characteristics of participating hospitals. Before the intervention period, PASI and DLQI were performed in 2 out of 3 participating centers, of which one center had automated PASI and DLQI calculations available in the electronic health record. DR was already performed, but not on a standard(ized) basis. No local protocols were available. Two hospitals reported initiating DR when patients had sustained low disease activity for at least 6 months.

#### Implementation fidelity

All components of the implementation strategy (Figure 2) were delivered to participating sites. Education meetings (T0) were provided on site for hospital 1 and 3, and online for hospital 2. A total number of 5 meetings were proposed (Figure 2), but short additional online meetings were scheduled for each center to discuss the local workflow, resulting in 6 meetings per site. The research team provided additional support by e-mail. HCPs primarily involved in psoriasis care and responsible for the execution of the study participated in all meetings and in individual interviews. Other HCPs from participating centers were updated in regular team meetings by colleagues involved in the project. After the first feedback meeting (T3), hospital 1 reported to have used all provided tools, hospital 2 only used the protocol, patient information leaflet, and administration text, and hospital 3 used the summary card and patient information leaflet. As not all provided tools were used, fidelity was less than 100%.

#### Feasibility of the implementation process

At feedback meetings (T3, T6), HCPs indicated feasibility of implementation of the DR protocol, but extra time for patient education and adjusting prescriptions was sometimes needed. Moreover, adjustment of proposed protocols to the local situation and dissemination at the local workplace required some time investment for involved HCPs. In hospital 2, performing PASI and DLQI was not always possible due to a lack of time and lack of options to calculate scores within the used electronic health record system. It was however described in words whether patients had low disease activity in the individual health record. For hospital 1, DR was mainly applied to patients on ustekinumab, as those patients visited the hospital more frequently on a clustered outpatient clinic compared to patients on adalimumab or etanercept.

#### Interviews with involved healthcare providers

Ten HCPs (Table 2) participated in individual interviews between January and April 2022 (T6). The (sub)themes developed in the qualitative analysis with corresponding illustrative quotes are presented in Table 3. Main themes were divided into barriers and facilitators to the implementation of biologic DR.

**Table 2.** Summarized characteristics of involved healthcare providers.

Characteristic	Total *N* = 10
Sex (female)	8
Age (years), median (range)	58.5
Profession	
Dermatologist	3
Nurse practitioner	3
Nurse	2
Medical assistant	2
Professional experience (years)	
5–10	2
10–15	1
15–20	2
>20	5
Experience with biologic treatment (years)	
0–5	2
5–10	1
10–15	2
15–20	5
Experience with biologic DR (yes)	7

Data are presented as N unless otherwise indicated. Abbreviations: DR: dose reduction.

**Table 3.** Overview of (sub)themes and corresponding quotes resulting from the interviews.

	Theme	Subtheme	Quote
**Barriers**	Healthcare providers’ barriers	Lack of awareness and familiarity	*“Everyone knows about the DR protocol, but from experience we have learned that it declines, specifically with the dermatologists. Therefore, I recall it from time to time, for example within staff meetings.” [HCP9]*
		Lack of knowledge and disbeliefs about DR	*“At first, I thought it to be a large step for those patients: how will I explain this to patients if I think to myself that it won”t be possible? You will convey these feelings to your patients somehow.” [HCP4]*
	Patients unwillingness to try DR	Positive experiences with biologic treatment	*“Effects are so good, and they have so little side effects of the medication, that”s the largest advantage of biologics over for example methotrexate. And they are so happy with that.” [HCP2]*
		Patients’ disbeliefs about DR	*“Look, there are patients who are saying: this might be a financial advantage for the hospital. Then I just think, well yes, it is an advantage for the hospital but it is also beneficial for yourself.” [HCP7]*
		Fear of disease flares	*“People who don”t dare it at all; you can”t convince them. They are so afraid to get back to the situation of having so much limitations in daily life. They just don”t dare it.” [HCP2]*
	Practical issues	Incompatibility of the DR protocol with current practice	*“Patients visit the doctor or the nurse practitioner at least once a year, but at the doctors” consultation, PASI or DLQI scores are not performed. It”s just because all patients are scattered across different locations and different colleagues, DR is more difficult to apply.” [HCP1]*
		Complexity of the DR protocol	*“We then just check with the patient how they think it”s going, what they think themselves, and if you see during the physical examination that the psoriasis is cleared, you can decide to start dose reduction.” [HCP3]*
		Lack of time	*“The consulting hours are very busy and you have different patients every 10 min. The challenge is to sit down and really take the time to explain these things.” [HCP6]*
		Lack of available resources	*“Your system must be in order to make sure you won”t lose time when processing PASI and quality of life scores, that kind of things. That it just works efficiently, that”s the most important thing because then it will be possible to integrate it into your consultations.” [HCP2]*
**Facilitators**	Healthcare providers’ facilitators	Positive beliefs in the concept of DR	*“Less injections are beneficial for patients. Furthermore, dose reduction will reduce costs, which is a good thing as consequently, we can keep prescribing biologics for patients who need them. [….] Yes, I can only see advantages of dose reduction.” [HCP5]*
		Awareness and familiarity with DR	*“If you will just keep informing each other and everyone will keep paying attention to dose reduction, then I think it will be possible. The whole team, all involved colleagues and dermatologists should stay involved.” [HCP8]*
		Access to knowledge and education	*“A clear explanation on beforehand is very important. It will make sure that you will be convinced about dose reduction yourself. This is important, as patients will feel it when you will not be convinced yourself. So I think that understanding dose reduction and being convinced about it yourself is very important.” [HCP4]* *“Such a protocol as was proposed, maybe with reminders or mentioning it again in another staff meeting in order to get the information into everyone”s heads again. Or through an article in our dermatologists” magazine, or at conferences. Yes, those should be the communication channels I think. Or maybe also a webinar.” [HCP6]*
	Availability of assistance for healthcare providers	Availability of tools and guidance	*“Well, it helps when you are having clear guidance on how to apply dose reduction. And that it is nationally acknowledged, so that you are not doing it randomly.” [HCP8]*
		Available staff for support	*“From experience, educating patients can best be delegated to nurse practitioners or nurses. You can shortly address dose reduction to patients and the nurse will do the refinements. It just takes too much time. Patients may have a lot of questions and before you know it 10 min will be gone, while you already are running out of time.” [HCP6]*
		Availability of resources	*“What helps is when your system works well, in a way that you can just work efficiently.” [HCP2]*
	Providing support for patients	Patient involvement in the decision-making process	*“In one consultation, we prepare patients toward dose reduction. As such, they will have the possibility to think about it and they will get used to it. Within the next visit, we will start with dose reduction. As such, patients can get used to the idea.” [HCP10]*
		Patient education	*“We have leaflets on our desks and at the time of discussing dose reduction I present the leaflet to the patient. You can also point out the steps. It is just a really clear description and also measurable. Maybe it sounds stupid, but it”s not a black-and-white copy but in real colors, that may also play a role. Speaking for myself, a colorless copy will provide less confidence than a clear leaflet in full color.” [HCP1]*
		Patients positive attitudes toward DR	*“And eventually, everyone will feel the need to use less medications, that is some kind of a standard opinion in my experience. People want to take as little as possible.” [HCP2]*

Abbreviations: DR: dose reduction; HCP: healthcare provider.

Participating HCPs reported a lack of routine and experience with DR and a lack of knowledge on DR as barriers to implementation of biologic DR. Education, awareness and familiarity could act as enhancers. Participating HCPs valued the developed tools, as these tools provide support for the clinician and the patient, make DR feasible and ensure that DR is applied more frequently. Repeated education and participation in research projects about DR could facilitate further implementation of biological DR according to participants. They suggested that besides the provided information on DR, further and future access to knowledge remains important and could be achieved by means of scientific publications, conference presentations, and the uptake of biologic DR in treatment guidelines.

Among factors influencing implementation of biologic DR according to involved HCPs were also patient-related factors. Participants reported that disbeliefs about DR among patients, fear of disease flares, and positive experiences with biologics (e.g., no side effects and high effectiveness) could contribute to patients’ unwillingness to try DR. Sufficient patient education and involvement of patients in decision-making were suggested as facilitators by participating HCPs. They also reported that the provided patient information leaflets were useful for patients. Moreover, it was suggested that informing patients about DR at start of biologic treatment might be helpful, as well as providing information in one consultation and initiating DR at the next consultation. During DR, offering healthcare access and communication or tools about new dosing schedules seemed important for patients according to involved HCPs.

Complexity of the DR protocol and incompatibility with current practice were identified factors that could limit implementation. It was mentioned that performing PASI and DLQI is more difficult than globally estimating disease activity and DR eligibility, specifically when scores cannot easily be processed within electronic health records. Performing the scores requires some time-investment and change of practice when not performed on a regular basis yet. Besides the scores, extra time might be needed for patient education and for modifying prescriptions. Among facilitators to overcome these barriers was the availability of staff for support. In case dermatologists are lacking time for installation of DR, it was suggested that other staff members such as nurses could perform patient education and clinical measurements. Furthermore, it was brought up that IT solutions such as availability of an automated PASI and DLQI scoring system and an automated decision aid within the electronic health record for checking DR eligibility could be useful. Regarding proposed dosing schedules, some participants reported that the DR steps for ustekinumab were too large (e.g., from 12 weeks to 18 weeks and subsequently to 24 weeks) or steps were taken too soon, as it might be difficult to motivate patients toward these large steps. As such, intermediate steps were preferred by participants.

### Part 2. Effect evaluation

**Figure 3.**
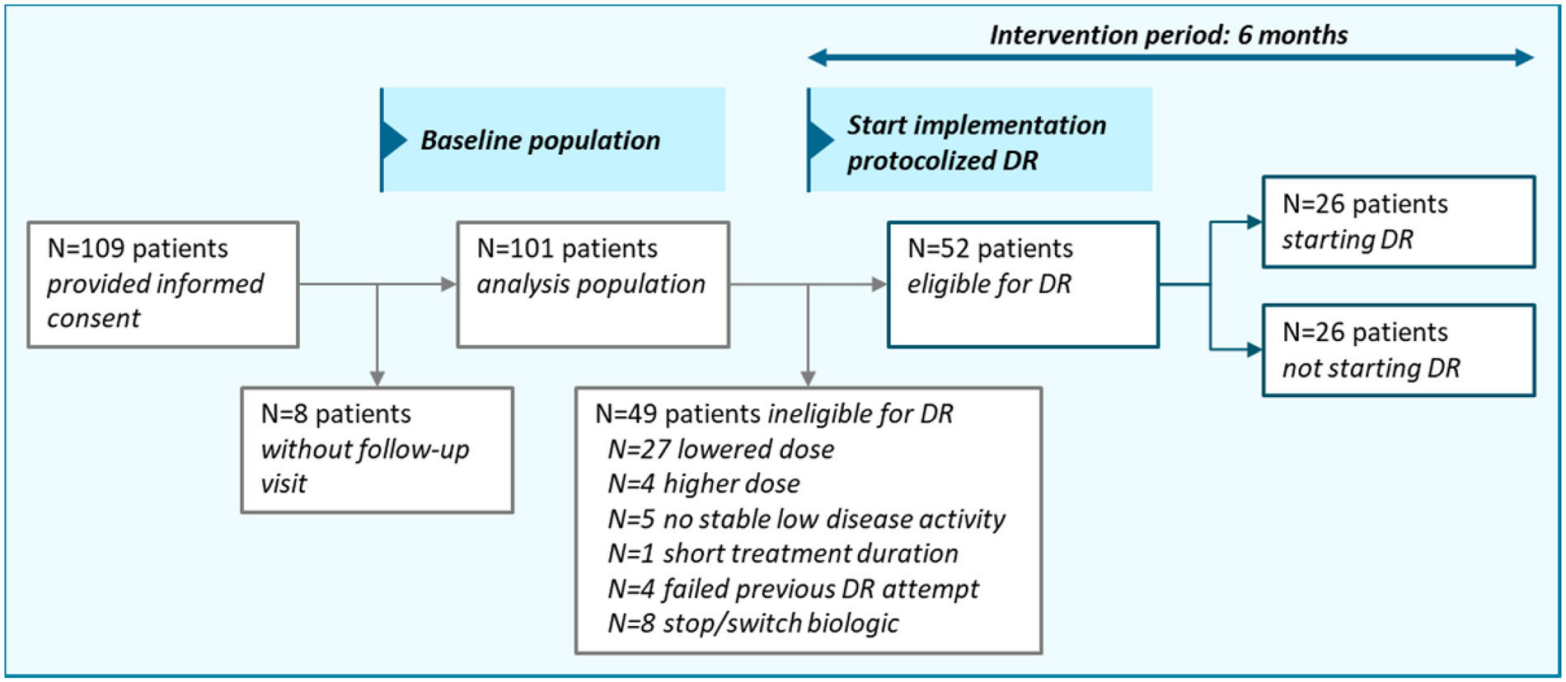
Flow chart of included patients, patients eligible for DR and patients starting DR during the intervention period. Patient data were collected in 2 hospitals. Abbreviations: DR: dose reduction. DR eligibility was based on the following criteria: plaque psoriasis, sustained low disease activity ≥6 months, use of the standard maintenance dose ≥6 months, low impact of psoriasis on patients’ dermatology-related quality of life, no failed previous DR attempt.

Patient characteristics and outcomes split per participating hospital are presented in Table S2. For the effect evaluation, 109 patients from 2 participating hospitals provided informed consent for data collection from their medical records. As in 8 patients no follow-up visits were available, 101 patients were included for analysis. See Figure 3 for a graphical overview.

At baseline (e.g., before the start of the intervention period), 49 of 101 included patients (48.5%) were ineligible for DR, due to the fact that they already used a lowered dose (*N* = 27), or did not fulfill the criteria for DR (*N* = 18), or failed a previous attempt for DR (*N* = 4) (Figure 3). During the intervention period of 6 months, 52 of 101 included patients (51.5%) were eligible for DR. In total, *N* = 26 (50% of eligible patients) started with DR during the intervention period. The other 26 eligible patients did not start DR. Available reasons for not starting DR were: patients being afraid of psoriasis flares (*N* = 2), experiencing increased psoriasis or itch at end of injection interval (*N* = 2), and not willing to start DR during the winter period (*N* = 1).

The proposed DR protocol was followed in 22 out of 26 patients (84.6%) who started DR during the intervention period. Four patients used other ustekinumab dosing schedules and one patients also not reached stable low disease activity for 6 months yet but did start DR. In hindsight, 12 out of 27 patients (44.4%) who already used a lowered dose at baseline followed the protocol. After the intervention, considerably more outcome measures (PASI, DLQI) were performed than at baseline of the intervention (Table S2). However, scores were still not measured in all patients on DR. Of note, according to the protocol, DLQI was not a prerequisite but was recommended to be performed apart from PASI.

## Discussion

This study aimed to investigate the implementation of protocolized DR of adalimumab, etanercept, and ustekinumab for patients with psoriasis in 3 general hospitals. Evaluation of the implementation process showed that a multicomponent implementation strategy including education for HCPs and provision of tools for assistance promoted uptake of protocolized DR. Different components of the implementation strategy were executed as planned and after the intervention, the proposed DR protocol was followed in the majority of patients that initiated DR.

Several additional factors for optimizing implementation of biologic DR were identified from interviews with involved HCPs. Among these factors was the need for staff for support, such as nurses who can provide patient education on DR. Another identified factor that could assist HCPs when performing DR was the availability of effective solutions within electronic health record systems such as decision aids or reminders together with the possibility for calculating scores (e.g., PASI and DLQI). This finding corresponds with previous rheumatological studies, which reported that issues with electronic health records could limit the uptake of dose optimization strategies, and providing treatment advice within electronic health records resulted in increased adherence to such a strategy (24,25). Besides these organizational factors, factors arising at the patients’ level were deemed important as well according to involved HCPs. HCPs suggested that informing patients at the treatment start, performing shared-decision making, and providing support by offering healthcare access and tools for new dosing schedules could be of added value for further uptake of protocolized biologic DR. Addition of these organizational and patient-related factors to our developed implementation strategy could as such promote further implementation of biologic DR.

Our effect evaluation of uptake of the DR protocol in daily practice revealed that after the intervention, the proposed DR protocol was followed in most patients on DR. Our data suggest that the implementation strategy resulted in increased numbers of patients on protocolized DR per timespan. During the relatively short intervention period of 6 months, 26 patients (25.7% of the total population) started DR. Before the intervention period, 27 patients were on a lowered dose, but this time period covered a maximum of one year before the intervention period, resulting in a larger time-window in which DR could have been initiated. Although we were not able to calculate the numbers of patients initiating DR per time unit, this indicates that our intervention led to a sharp increase of patients starting DR within 6 months. Due to the fact that some active patients had not visited the outpatient clinic during the intervention period, the total number of patients that could reduce their dose could have been higher with a longer follow-up. Among eligible patients were however patients unwilling to start DR. We also have previously demonstrated that a number of patients with psoriasis might not be willing to initiate DR due to fear of disease flares (9). This emphasizes that patient education is an important target for effective implementation, as information on the actual (low) risk of DR failure and the high probability of regaining low disease activity afterward is relevant for patients to balance the risks and benefits of DR (1,26,27).

As stated above, the proposed DR protocol was followed by HCPs in the majority (85%) of patients on DR. However, practice was heterogeneous across participating hospitals and clinical scores were not always performed. For ustekinumab, alternative dosing schedules were used sometimes, mostly consisting of subsequent injection interval prolongations of 2 weeks. HCPs indicated several barriers to the performance of the scores, including a lack of time and options to process scores within used electronic health record systems. The latter may explain why clinical scores were less frequently measured in hospital 2, where no automated calculations were available within the electronic health record system. Time constraints have previously been identified as a barrier for performing biologic DR in daily psoriasis care (3,6,9). However, in the light of potential cost savings resulting from DR, the question arises if HCPs should be given extra time in order to apply DR. Another possible solution would be the reported availability of staff for support of dermatologists or efficient IT solutions. It can however also be debated if more flexible approaches to the use of DR eligibility criteria should be allowed. However, use of clear criteria could enable timely actions to prevent disease flares (26). Involved HCPs also reported that initiating DR based on outcome measures (e.g., PASI, DLQI) is more objective and transparent for both patients and clinicians, and scores make it possible to compare assessments between consultations. A possible solution here would be the use of less time-consuming outcome measures such as Physician Global Assessments (PGA) of disease severity.

A strength of our study is the combined evaluation of the implementation process itself and the actual uptake of protocolized biologic DR in clinical practice. Data were collected using both quantitative and qualitative methods, which broadened our findings. Combining both methods can provide unique insights in multifaceted phenomena such as implementation processes. In our study, qualitative analysis of interviews with involved HCPs enabled an in-depth evaluation of the implementation process and provided insight into relevant factors outside the targeted components of our implementation strategy. For future research, it could be considered to use focus groups instead of individual interviews in order to explore interactions between participants and elaborate on solutions for identified barriers (28). We developed a multicomponent implementation strategy that targeted several possible barriers for change. Such multicomponent strategies are more effective than simple approaches for the implementation of innovations in healthcare (14,15). Our used implementation components and identified additional factors influencing effective implementation of protocolized DR could help inform future efforts to implement DR on a larger scale.

This study also has its limitations. First, the implementation strategy was tested in a specific, national setting. This could influence generalizability to other healthcare systems as results of implementation processes are dependent of organizational and contextual aspects (13). Second, our intervention period was relatively short. This might have limited the actual uptake of the DR protocol and resulted in a possible underestimation. As some patients had no follow-up visit available, DR could not yet have been initiated in these patients. Third, due to the uncontrolled design, we were not able to conclude which part of our strategy was most effective nor to define a causal relationship between the intervention and results as theoretically, other factors in the intervention period could have influenced results. Additionally, not all provided tools were used across participating hospitals. Last, we were only able to analyze patient data of patients who provided informed consent, resulting in a response rate of approximately 30%. Hence, analyses were explorative and no precise estimations of differences between before- and after measures could be made.

In conclusion, results of our pilot study demonstrated feasibility of a strategy to implement protocolized biologic DR for patients with psoriasis in daily dermatological practice. Provision of protocols, patient information leaflets, and education for HCPs were important tools for implementation of DR, and led to an increase of patients that underwent protocolized DR of the biologics adalimumab, etanercept or ustekinumab. Important factors for further dissemination of protocolized biologic DR into practice may include the availability of additional staff for support of physicians and patients, extra time during consultations, uptake of DR into treatment guidelines, and effective tools such as feasible protocols and IT solutions. An integrated approach combining these relevant factors in the context of the targeted healthcare setting together with the involvement of relevant stakeholders could lead to increased numbers of patients on protocolized biologic DR. Eventually, this will lead to decreased long-term drug exposure for our patients and substantial cost savings (2,9).

## Supplementary Material

Supplemental Material

## Data Availability

The data that support the findings of this study are available from the corresponding author upon reasonable request.
