# Supplementary material for: Steps towards implementation of protocolized dose reduction of adalimumab, etanercept and ustekinumab for psoriasis in daily practice
Source: J Dermatolog Treat. 2023 Mar 13;34(1):2186728. doi: 10.1080/09546634.2023.2186728 (PMC10013325; doi:10.1080/09546634.2023.2186728)
Supplement: Supplemental Material [file IJDT_A_2186728_SM0516.pdf]

Supplemental Material

Steps towards implementation of protocolized dose reduction of adalimumab, etanercept and ustekinumab for psoriasis in daily practice

L.S. van der Schoot[a], J.J. Janssen[a], M.T. Bastiaens[b], A. de Boer-Brand[c], C. Christiaansen-Smit[b], D.N.H. Enomoto[d], R. Hovingh[b], R.A. Tupker[c], M.M.B. Seyger[a], L.M. Verhoef[e], J.M.P.A. van den Reek[a], E.M.G.J. de Jong[a,f]

[a]Department of Dermatology, Radboud University Medical Center, Nijmegen, the Netherlands; [b]Department of Dermatology, Elisabeth-TweeSteden Ziekenhuis, Tilburg, the Netherlands; [c]Department of Dermatology, St Antonius Ziekenhuis, Nieuwegein, the Netherlands; [d]Department of Dermatology, Dermatologisch Centrum Isala, Zwolle, the Netherlands; [e]Department of Rheumatology, Sint Maartenskliniek, Nijmegen, the Netherlands; [f]Radboud University, Nijmegen, the Netherlands.

Content:

Figure S1. Patient information leaflet
Table S1. Implementation strategy: components and theoretically based barriers that are targeted
Table S2. Patient and treatment characteristics split per participating hospital

Figure S1. Patient information leaflet

Dose reduction of biologics in adult patients with moderate to severe psoriasis

You are treated with one of the following biologics for your psoriasis: adalimumab, etanercept, or ustekinumab.

What are biologics?

Biologics are medicines that have been designed to treat psoriasis. They work by specifically targeting proteins in the body which are involved in inflammation. By blocking these proteins, biologics can improve symptoms of psoriasis. Biologics may decrease the body's ability to fight infections. Among the most frequently reported side effect are mild infections such as cold and flu symptoms.

Dose reduction of biologics

The goal of dose reduction of biologics is to strive for the lowest but still effective dose. Previous research has shown that the dose can be reduced in a part of the patients with psoriasis, while the treatment effect remains good. With dose reduction, the risk of side effects will be reduced. The societal healthcare costs will also decrease with the application of dose reduction.

If your psoriasis is clear or almost clear for at least 6 months when using the standard dose, we will try to reduce the dose. The dose will be reduced by extending the time between the injections, in two steps. By reducing the dose carefully in two steps, you will eventually receive the lowest, effective dose. The biologic will not be stopped, because we know that psoriasis will then eventually return.

What if my psoriasis worsens?

If your psoriasis worsens when using a lowered dose or in case you do not feel comfortable with using the lowered dose, the dose can be increased or the standard dose can be resumed in consultation with your treating healthcare professional.

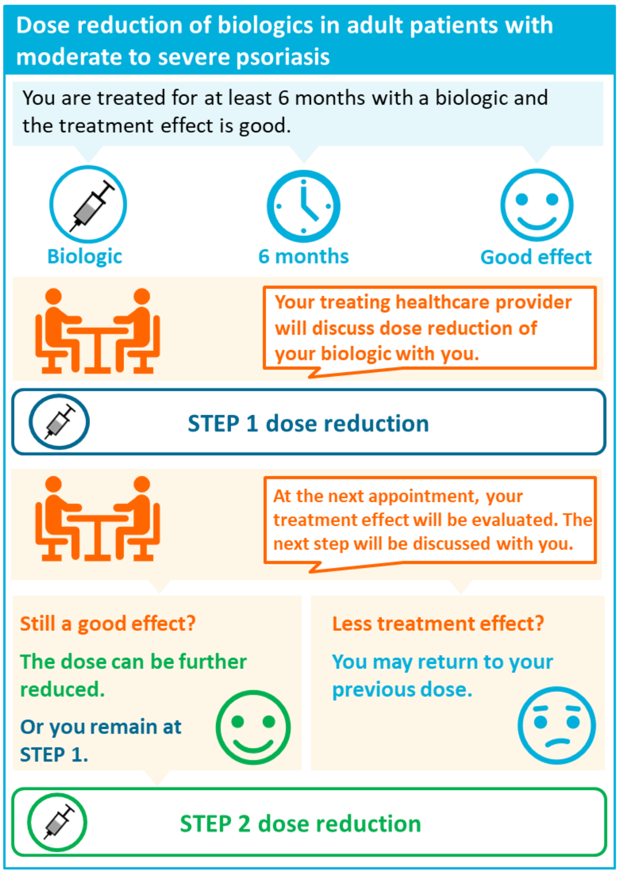

English translation of the used patient information leaflet. Of note, this leaflet is a generic example and does therefore not include dosing schedules per biologic. Within the implementation pilot, different versions per biologic were provided with inclusion of dosing schedules per biologic.

Table S1. Implementation strategy: components and theoretically based barriers that are targeted

Components	Barriers that are targeted [1]
**Inventory**<br>First meeting to make an inventory of the situation in the hospital and make a planning for the total project.	• Feasibility: local situation is specific for each hospital, tailoring is needed.<br>• Source of the recommendation: do the organisation(s) and people who made the recommendation have credibility with the targeted healthcare providers?<br>• Effort: what amount of effort is required to change or adhere?
**Education**<br>Distribution of relevant documents/articles, presentation with overview of the literature about tightly controlled biologic dose reduction in psoriasis patients.	• Lack of knowledge: tailored information or education that helps the targeted healthcare providers to fit the recommended behaviour into their current practice is needed.<br>• Lack of awareness and familiarity.<br>• Compatibility of recommended protocol with current practices.<br>• Lack of patients motivation: provide the targeted healthcare providers with aids or strategies to motivate patients.
**Protocols**<br>Development of, and agreement on, relevant local treatment protocols.	• Lack of assistance for clinicians: healthcare providers have no protocols to help them adhere to the recommendations.<br>• Feasibility: ensuring that clinical intervention is practical for optimal adherence.
**Feedback**<br>Feedback meetings including provision of advice, discussing local workflows, and involved healthcare providers' feedback on the implementation process after 3 and 6 months.	• Lack of insight into own practice, incl. visibility of benefits of the new strategy.

References

1. Flottorp SA, Oxman AD, Krause J, et al. A checklist for identifying determinants of practice: a systematic review and synthesis of frameworks and taxonomies of factors that prevent or enable improvements in healthcare professional practice. Implement Sci. 2013 Mar 23;8:35.

Table S2. Patient and treatment characteristics split per participating hospital

	Hospital 2	Hospital 3	Total
N (% of total number of participants)	55 (50.5)	54 (49.5)	109
Baseline characteristics			
Sex (female)	21 (38.2)	23 (42.6)	44 (40.4)
Age (years), median (IQR)	57 (46 – 68)	58 (49 – 65)	58 (47.5 – 66)
BMI (kg/m^2), median (IQR)	27 (24.5 – 29.4)[a]	29 (26.1 – 34.9)[b]	27.6 (25.1 – 30.5)[c]
Disease duration (years)	16 (13 – 26)[d]	27 (16.3 – 40.7)[e]	20 (14 – 33)[f]
Psoriasis subtype (current)			
Plaque psoriasis	53 (96.4)	46 (85.2)	99 (90.8)
Plaque psoriasis and other subtype	2 (3.6)	4 (7.4)	6 (5.5)
Other subtype[g]	0	4 (7.4)	4 (3.7)
Comorbidities			
Psoriatic arthritis	14 (26.4)[h]	11 (20.4)	25 (22.9)[i]
Other spondyloarthropathy	0	1 (1.9)	1 (0.9)
Hidradenitis suppurativa	0	0	0
Inflammatory bowel disease	2 (3.6)	0	2 (1.8)
History of previous biologic use (yes)	19 (34.5)	23 (42.6)	42 (38.5)
Current treatment			
Adalimumab	44 (80)	21 (38.9)	65 (59.6)
Etanercept	3 (5.5)	5 (9.3)	8 (7.3)
Ustekinumab	8 (14.5)	28 (51.9)	36 (33)
Treatment duration (years), median (IQR)	3.1 (1 – 8.3)	5.6 (2.5 – 8.7)	4 (1.4 – 8.3)
Previous DR			
Yes, successful	11 (20)	18 (33.3)	29 (26.6)
Yes, unsuccessful	5 (9.1)	17 (31.5)	22 (20.2)
No, not discussed	28 (69.1)	19 (35.2)	57 (52.3)
No, patient was not willing	1 (1.8)	0	1 (0.9)
Comedication			
Methotrexate	0	1 (1.9)	1 (0.9)
Acitretin	0	1 (1.9)	1 (0.9)
Prednisolone	0	1 (1.9)	1 (0.9)
None	0	51 (94.4)	106 (97.2)
Patients on lowered dose at baseline	8 (14.5)	19 (35.2)	27 (24.8)
Adalimumab[j]	8 (18.2)	6 (28.6)	14 (21.5)
Etanercept[j]	0	1 (20)	1 (12.5)
Ustekinumab[j]	0	12 (42.9)	12 (33.3)
Outcome measures used (patients on lowered dose)			
DLQI	0	14 (73.7)	14 (41.9)
PASI	0	8 (42.1)	8 (29.6)
Effect evaluation outcomes (intervention period)			
Patients starting DR	14 (25.4)	12 (22.2)	26 (23.9)
Adalimumab[j]	12 (27.3)	6 (28.6)	18 (27.7)
Etanercept[j]	1 (33.3)	0	1 (12.5)
Ustekinumab[j]	1 (12.5)	6 (21.4)	7 (19.4)
DR protocol followed (patients on DR)[k]	14 (100)	8 (66.7)	22 (84.6)[l]
Outcome measures used (patients on DR)			
DLQI	11 (78.6)	10 (83.3)	21 (80.8)
PASI	6 (42.9)	6 (50)	12 (46.2)

Data are presented as N (%) unless otherwise indicated. Abbreviations: BMI, body mass index; DR, Dose Reduction; DLQI, Dermatology Life Quality Index; IQR, interquartile range; PASI, Psoriasis Area and Severity Index. Missing data (N): [a]22, [b]46, [c]68, [d]12, [e]18, [f]30, [h]2, [i]2.

[g]Reported psoriasis subtypes included inverse psoriasis, nail psoriasis, palmoplantar psoriasis, and palmoplantar pustulosis.

[j]Proportions were calculated based on numbers of patients per biologic.

[k]DR protocol was followed when proposed dosing schedules were used and when criteria were met.

[l]N=4 patients used other DR dosing schedules and N=1 also not reached stable low disease activity for 6 months yet but did start DR. In case PASI scores were not performed it had to be reported that psoriasis was (almost) clear in order to follow the proposed protocol.
